# Sensitization of MCF7 Cells with High Notch1 Activity by Cisplatin and Histone Deacetylase Inhibitors Applied Together

**DOI:** 10.3390/ijms22105184

**Published:** 2021-05-13

**Authors:** Anna Wawruszak, Jarogniew Luszczki, Marta Halasa, Estera Okon, Sebastian Landor, Cecilia Sahlgren, Adolfo Rivero-Muller, Andrzej Stepulak

**Affiliations:** 1 Department of Biochemistry and Molecular Biology, Medical University of Lublin, 20-093 Lublin, Poland; marta.halasa@umlub.pl (M.H.); estera.okon@umlub.pl (E.O.); adolfo.rivero-muller@umlub.pl (A.R.-M.); andrzej.stepulak@umlub.pl (A.S.); 2 Department of Pathophysiology, Medical University, 20-090 Lublin, Poland; jarogniew.luszczki@umlub.pl; 3 Faculty of Science and Engineering, Cell Biology, Åbo Akademi University, 20500 Turku, Finland; sebastian.landor@abo.fi (S.L.); cecilia.sahlgren@abo.fi (C.S.); 4 Turku Bioscience Centre, Åbo Akademi University and University of Turku, 20500 Turku, Finland; 5 Institute for Complex Molecular Systems, Eindhoven University of Technology, 5612 Eindhoven, The Netherlands

**Keywords:** breast cancer, Notch1, histone deacetylase inhibitors (HDIs), valproic acid (VPA), vorinostat (SAHA), cisplatin (CDDP), isobolographic analysis

## Abstract

Histone deacetylase inhibitors (HDIs) are promising anti-cancer agents that inhibit proliferation of many types of cancer cells including breast carcinoma (BC) cells. In the present study, we investigated the influence of the Notch1 activity level on the pharmacological interaction between cisplatin (CDDP) and two HDIs, valproic acid (VPA) and suberoylanilide hydroxamic acid (SAHA, vorinostat), in luminal-like BC cells. The type of drug–drug interaction between CDDP and HDIs was determined by isobolographic analysis. MCF7 cells were genetically modified to express differential levels of Notch1 activity. The cytotoxic effect of SAHA or VPA was higher on cells with decreased Notch1 activity and lower for cells with increased Notch1 activity than native BC cells. The isobolographic analysis demonstrated that combinations of CDDP with SAHA or VPA at a fixed ratio of 1:1 exerted additive or additive with tendency toward synergism interactions. Therefore, treatment of CDDP with HDIs could be used to optimize a combined therapy based on CDDP against Notch1-altered luminal BC. In conclusion, the combined therapy of HDIs and CDDP may be a promising therapeutic tool in the treatment of luminal-type BC with altered Notch1 activity.

## 1. Introduction

Female breast cancer (BC) was the leading cause of cancer incidence globally in 2020. It accounted for 11.7% of all cancer cases with approximately 2.3 million new cases among women during the last year alone. It was also the fifth leading cause of cancer mortality, with 685,000 deaths worldwide [1].

BC is a heterogeneous disease with variable biological behavior, morphological features, and response to therapy. The current routine histological analyses for detection of the presence or absence of estrogen receptor (ER), progesterone receptor (PR), and human epidermal growth factor receptor 2 (HER2) represent the earliest attempts to provide a more personalized approach to BC therapy, based on molecular drivers of the disease [2].

Luminal BC is hormone-receptor positive (ER+ and/or PR+), further subdivided into luminal A subtype characterized by low (<20%) expression of the Ki-67 protein (a proliferation index marker) and lack of HER2, and luminal B subtype, which is HER2 positive with over 20% cells expressing Ki-67 protein. Patients harboring luminal BC have better prognosis than women diagnosed with other BC subtypes [3,4]. Although hormone therapy is standard management of BC patients with ER and/or PR expressing tumors, multi-drug resistance or ER acquired mutations are serious clinical problems that limit the use of standard therapeutic options (adriamycine, paclitaxel) [5]. Therefore, new effective therapeutic options including cisplatin (CDDP), which is currently applied for TNBC (triple negative: ER-/PR-/HER2- BC) tumors, are considered for the treatment regimens of patients with luminal BC [6]. The mechanism of action of CDDP is associated with ability to create a crosslink with the purine bases on the DNA structure to form DNA adducts, which prevents repair of the DNA leading to DNA damage, and subsequently induces apoptosis in cancer cells. Unfortunately, CDDP has many side effects including nausea, cardiotoxicity, nephrotoxicity, neurotoxicity, or hepatotoxicity. Due to these complications, other anti-cancer drugs are used in combination with CDDP to achieve better clinical response along with reduced CDDP doses and, thus less toxic side effects and/or ameliorated drug resistance (sensitization) [7].

Histone deacetylases (HDACs) are a family of epigenetic enzymes that remove acetyl (Ac) groups from histones and non-histone proteins. Acetylation of proteins changes their function, localization, and stability. Acetylation of histones is commonly associated to open chromatin and increased gene transcription [8]. Cancer cells have different epigenetic landscape than normal cells, where normally repressed oncogenes, or genes related to drug resistance, are expressed, while tumor suppressors are repressed instead [8].

Inhibitors of histone deacetylases have been used in the clinic for more than 40 years in epilepsy [9] but have also found to be anti-neoplastic agents versus several cancer types [8]. Histone deacetylase inhibitors (HDIs) are currently being tested in BC as single agents or in combination, providing very promising results both in vitro and in vivo [10], as well as in clinical trials [11,12,13,14].

Suberoylanilide hydroxamic acid (SAHA) is HDI approved by the Food and Drug Administration (FDA) for patients with cutaneous T-cell lymphoma. It has been demonstrated that SAHA enhances radiosensitivity and suppresses lung metastasis in BC both in vitro and in vivo. Interestingly, data from clinical trials showed that SAHA can be well tolerated and demonstrates limited toxicity, which is rapidly reversible upon discontinuation of the drug [15]. Valproic acid (VPA) is HDI belonging to the group of short-chain fatty acids. It has been approved for the treatment of epilepsy and bipolar disorder. Currently, VPA is being examined in a large number of clinical trials for different types of leukemia and solid tumors. It has been demonstrated that VPA is a selective anti-proliferative agent in estrogen-sensitive BC [16]. All these findings suggest that both SAHA and VPA alone or in combination with ionizing radiation (IR), as well as with other chemotherapeutic agents, could serve as potential therapeutic strategies for BC patients [15].

Recent studies have also demonstrated that VPA and SAHA can affect Notch signaling in cancers [17,18]. In the Notch signaling, a Notch trans-membrane receptor (Notch 1 to 4) interacts extracellularly with one of the ligands (Jagged 1,2, delta-like 1, 3, 4) on a contacting cell, initiating proteolytic cleavage of the receptor and the subsequent release of the Notch intracellular domain (NICD), which then translocates to the nucleus where it interacts with the DNA binding protein CBF1/Su(H)/Lag-1 (CSL, also known as RBPJ) [19]. Four Notch paralogs play different roles in the formation and development of BC. It has been noted that high level of Notch1, Notch3, and/or Notch4 expression is associated with poor prognosis and clinical outcome of BC patients. In contrast, Notch2 acts as a tumor suppressor [18,20]. The exact role of different Notch isoforms in BC is yet to be defined. However, Notch1 seems to be the most pivotal for BC progression. It has also been demonstrated that increase of Notch1 expression correlates with a dramatic reduction of the overall survival of patients suffering BC [18,21]. 

In our previous study, we determined the influence of SAHA and VPA, individually or in combination with CDDP, on Notch signaling in TNBC. We also demonstrated additive type of pharmacological interactions between CDDP and SAHA or VPA at the fixed-ratio combination of 1:1 in TNBC cancer cell lines with increased or decreased Notch1 activity [18]. MTT [3-(4,5-dimethylthiazol-2-yl)-2,5-diphenyltetrazolium bromide] assay was used to evaluate antiproliferative effect of HDIs/CDDP treatment in the BC in vitro models, although previously [22,23] we showed by other methods the results of these drugs individually or/and in combinations on apoptosis [22], cell cycle arrest [22], and BrdU incorporation [23].

There is no publication regarding the effects of either SAHA or VPA, individually or in combination with other cytostatic drugs, on the Notch signaling pathway in BC across the entire Pubmed database. The only article on this topic is our previous article in the context of TNBC. There is a huge knowledge gap in this area of research. Due to the fact that no data are available on whether Notch activity has an impact on the success or failure of received treatment in patients with luminal-type BC, in our current study we examined whether reduction or increase in Notch1 activity affects the HDIs/CDDP drug–drug interactions.

## 2. Results

### 2.1. Expression and Activity of Notch1

Two MCF7 cell lines with high (Notch1^high^MCF7) or low (Notch1^low^MCF7) Notch1 activity were created by stably transfecting either the ΔEN1ICD, a truncated Notch1 that is immediately cleaved at the membrane releasing N1ICD, or the dominant negative CSL (dnCSL), a cytoplasmic CSL that sequesters any active NICD before it can translocate to the nucleus, respectively [18,24]. Notch1^high^MCF7 cells expressed higher level of NICD, while the Notch1^low^MCF7 cell line had a similar NICD level as wild-type (WT) parental MCF7 cells (native), which was previously determined by immunoblotting [24]. Notch1^high^MCF7 cells had high Notch activity, as analyzed by a reporter assay, while Notch1^low^MCF7 had lower Notch1 activity than native cells [24]. 

### 2.2. Dose-Dependent Growth Inhibition of Native and Transfected MCF7 Breast Cancer Cells after CDDP and HDIs Treatment

**Figure 1:**
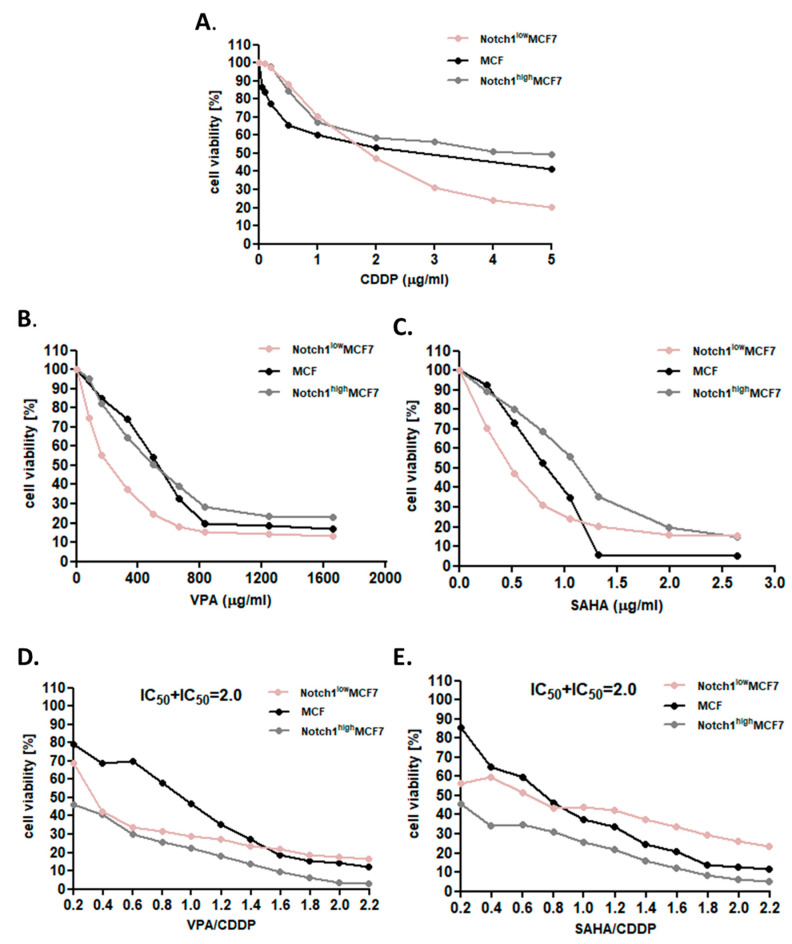
The anti-proliferative effects of CDDP, VPA, or SAHA in MCF7 cells. (**A**) The anti-proliferative effect of CDDP in MCF7 [22], Notch1^low^MCF7, and Notch1^high^MCF7 cells; (**B**) the anti-proliferative effect of VPA in MCF7 [22], Notch1^low^MCF7, and Notch1^high^MCF7 cells; (**C**) the anti-proliferative effect of SAHA in MCF7 [22], Notch1^low^MCF7, and Notch1^high^MCF7 cells; (**D**) the anti-proliferative effect of combined treatment of VPA and CDDP in MCF7 [22], Notch1^low^MCF7, and Notch1^high^MCF7 cells; (**E**) the anti-proliferative effect of combined treatment of SAHA and CDDP in MCF7, Notch1^low^MCF7, and Notch1^high^MCF7 BC cells. Transformed and native MCF7 cells were exposed to concomitant HDIs’ and CDDP treatment using different ratios of the IC_50_ (2.0 = IC_50_ + IC_50_). The cell viability was measured by the MTT assay. The results are presented from three independent experiments (*n* = 18).

The anti-proliferative effect of CDDP, VPA, and SAHA was analyzed in MCF7 cells with increased and decreased Notch1 activity, versus native cells, using the MTT (3-(4,5-dimethylthiazol-2-yl)-2,5-diphenyltetrazolium bromide) assay in order to establish the IC_50_ value for each analyzed compound in all lines (Table 1). In the study, we revealed the growth inhibition effect of each compound (CDDP, VPA, and SAHA) in all analyzed BC cell lines in dose-dependent fashion. The cytotoxic effect of CDDP was lower in MCF7 cells with altered Notch1 activity than in native cells in the lower concentrations (up to 2 µg/mL). In the case of VPA and SAHA, the cytotoxic effect was higher for cells with decreased Notch1 activity and lower for cells with increased Notch1 activity than for native cells (Figure 1). Decreased activity of Notch1 results in more than two-fold reduced doses of VPA or SAHA to achieve IC_50_ value in these cell lines (Figure 1 and Table 1). Then, we focused on the growth inhibition effect of the combination of CDDP and HDIs. In both cases, non-transfected native (or WT) cells treated with either CDDP and VPA or CDDP with SAHA in lower doses were more resistant than cells with altered Notch1 activity. In higher doses, Notch1^low^MCF7cells were more sensitive, whereas Notch1^high^MCF7cells were more resistant to CDDP/HDIs’ treatment than native cells. Interestingly, we observed changes in sensitivity to CDDP/HDIs’ treatment combination in Notch1^low^MCF7 and Notch1^high^MCF7 cells versus CDDP or HDIs used individually. All these results suggest that combinational treatment of CDDP and any of the tested HDI sensitizes MCF7 cells with high Notch1 activity to treatment. 

### 2.3. Effect of SAHA or VPA on the Anti-Proliferative Effects of CDDP in MCF7 cells with Increased Activity of Notch1 (Notch1^high^MCF7)

**Figure 2:**
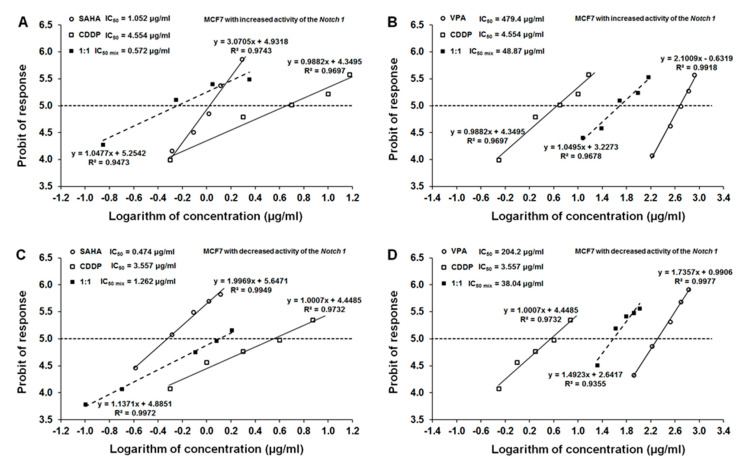
Log-probit dose-response relationship lines for CDDP, SAHA, and VPA in transfected MCF7 cells. (**A**) Log-probit dose-response relationship lines for CDDP and SAHA administered alone and in combination at the fixed-ratio of 1:1, with respect to their anti-proliferative effects on MCF7 cells with increased activity of Notch1 (Notch1^high^MCF7); (**B**) log-probit dose-response relationship lines for CDDP and VPA administered alone and in combination at the fixed-ratio of 1:1, with respect to their anti-proliferative effects on Notch1^high^MCF7cells; (**C**) log-probit dose-response relationship lines for CDDP and SAHA administered alone and in combination at the fixed-ratio of 1:1, with respect to their anti-proliferative effects on MCF7 cells with decreased activity of Notch1 (Notch1^low^MCF7); (**D**) log-probit dose-response relationship lines for CDDP and VPA administered alone and in combination at the fixed-ratio of 1:1, with respect to their anti-proliferative effects on MCF7 cancer cells with decreased activity of Notch1 (Notch1^low^MCF7). Doses of particular compounds (CDDP, SAHA, and VPA) administered both separately and in combination were transformed into logarithms, whereas the anti-proliferative effects produced by the drugs in the cancer cell line MCF7 were transformed into probits according to Tallarida method [25]. Equations of dose-response relationship lines are presented on the multipart figure. Respective IC_50_ values are depicted in the left corners of each panel.

The separate administration of CDDP, SAHA, or VPA resulted in a clear-cut anti-proliferative effect of the tested drugs in the MCF7 cells with increased activity of the Notch1 (Figure 2A,B). Linearly related dose-response (log-probit) effects allowed for the calculation of the IC_50_ values for CDDP, SAHA, and VPA that amounted to 4.554 ± 2.737 μg/mL, 1.052 ± 0.203 μg/mL, and 479.4 ± 135.5 μg/mL, respectively (Figure 2AB). All dose-response effect (log-probit) [25] lines between CDDP + SAHA and CDDP + VPA for the Notch1^high^MCF7 cells were not parallel to each other (Figure 2A,B).

### 2.4. Effect of SAHA or VPA on the Anti-Proliferative Effects of CDDP on MCF7 cells with Decreased Activity of Notch1 (Notch1^low^MCF7)

The single administration of CDDP, SAHA, or VPA resulted in a clear-cut anti-proliferative effect of the tested drugs on MCF7 cells with decreased activity of the Notch1 (Figure 2C,D). In the Notch1^low^MCF7, the IC_50_ values for CDDP, SAHA, and VPA were 3.557 ± 2.111 μg/mL, 0.474 ± 0.141 μg/mL, and 204.2 ± 69.86 μg/mL, respectively (Figure 2C,D). All dose-response effect (log-probit) lines between CDDP + SAHA or CDDP + VPA for Notch1^low^MCF7 were not parallel to each other (Figure 2C,D).

### 2.5. Type I Isobolographic Analysis of Interaction for the Combinations of CDDP with SAHA or VPA on Notch1^high^MCF7 Cells

**Figure 3:**
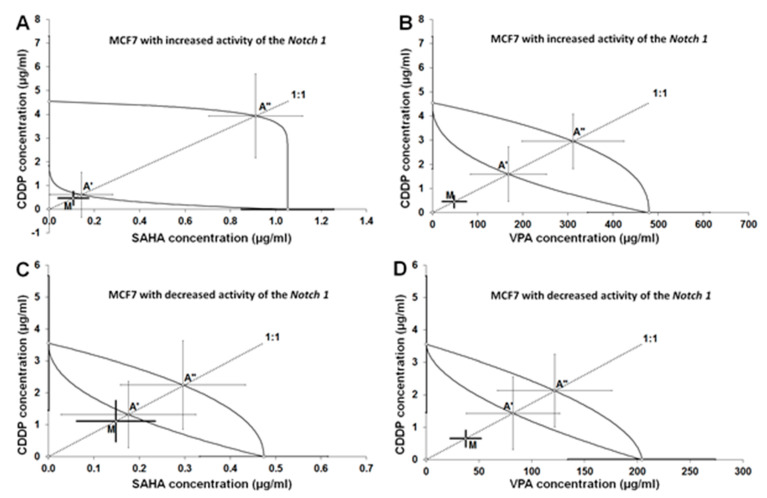
Isobolograms illustrating additive interactions between CDDP, SAHA, and VPA with respect to their anti-proliferative effects on MCF7 cells with increased (**A**,**B**) and decreased (**C**,**D**) Notch1 activity. Median inhibitory concentrations (IC_50_ ± S.E.M.) for CDDP, SAHA, and VPA are plotted graphically on the X-and Y-axes, respectively. The lower and upper isoboles of additivity represent the curves connecting the IC_50_ values for CDDP and SAHA or VPA administered alone. The dotted line corresponds to the fixed ratio of 1:1 for the combination of CDDP with SAHA or VPA. The points A’ and A” depict the theoretically calculated IC_50_ add values (± S.E.M.) for both lower and upper isoboles of additivity. The point M represents the experimentally derived IC_50 mix_ value (± S.E.M.) for total dose of the mixture that produced a 50% anti-proliferative effect in MCF7 cells. The experimentally derived IC_50 mix_ value is placed close to the point A’ for the lower isobole of additivity (**A**,**C**), indicating additive interaction between CDDP and SAHA or VPA. Although the experimentally derived IC_50 mix_ value is placed below the additive point A’ (**B**,**D**), no statistical significance was attained between CDDP and SAHA or VPA, indicating additive interaction with a tendency toward synergy in MCF7 cells.

The combinations of CDDP with SAHA or CDDP with VPA (both at the fixed ratio of 1:1) produced clear-cut anti-proliferative effects on the Notch1^high^MCF7. The experimentally determined IC_50_ mix values for the two-drug mixture were 0.572 ± 0.362 μg/mL (CDDP with SAHA; Table 2, Figure 3A) and 48.87 ± 27.65 μg/mL (CDDP with VPA; Table 1, Figure 3B). The type I isobolographic analysis for non-parallel dose-response effects revealed no statistical difference between the compared values (i.e., between the IC_50_ mix and IC_50_ add values) with unpaired Student’s *t*-test and, thus, the analyzed interaction between CDDP and SAHA was additive while that between CDDP and VPA was additive with a tendency toward synergy (Table 3).

### 2.6. Type I Isobolographic Analysis of Interaction for the Combinations of CDDP with SAHA or VPA on Notch1^low^MCF7 Cells

Likewise, the combinations of CDDP + SAHA or CDDP + VPA (at the fixed ratio of 1:1) produced the definite anti-proliferative effects in Notch1^low^MCF7 cells. The experimentally determined IC_50_ mix values for the two-drug mixture were 1.262 ± 0.737 μg/mL (CDDP with SAHA; Table 2, Figure 3C) and 38.04 ± 15.14 μg/mL (CDDP with VPA; Table 2, Figure 3D). With type I isobolographic analysis for non-parallel dose-response effects, no statistically significant difference was observed between the IC_50_ mix and IC_50_ add values (unpaired Student’s *t*-test; Table 2). Thus, lack of statistically significant difference confirmed that the analyzed interaction between CDDP + SAHA was additive (Figure 3C), while that of CDDP + VPA was additive with a slight tendency toward synergy (Figure 3D; Table 3).

### 2.7. Analysis of the Types of Pharmacological Interaction between CDDP and HDIs on Cells with Altered Notch1 Activity with Reference to Native MCF7 Cells

Isobolographic analysis of interaction for non-parallel DRRCs revealed that the mixture of CDDP with SAHA at the fixed ratio of 1:1 exerted an additive interaction both in the Notch1^high^MCF7 and Notch1^low^MCF7 lines (Table 3). A similar tendency was observed in the MCF7 cells co-treated with CDDP and SAHA as well as CDDP and VPA [22]. A better type of pharmacological interaction between CDDP and VPA was observed in the cells with altered Notch activity compared to the native cells. Additivity with a tendency toward synergy was observed for the combination of CDDP with VPA in Notch1^high^MCF7 and Notch1^low^MCF7 lines. Summarizing, SAHA and VPA might be considered as potential therapeutic agents in therapy with CDDP against receptors-positive BC with altered Notch1 activity (Table 3).

## 3. Discussion

The effectiveness of BC therapy using different modes of treatment with several anti-cancer drugs including classical chemotherapy, hormone, and targeted therapy tools has been extensively studied and discussed in the last decade [28]. However, despite the considerable progress that has been made in hormone-responsive breast cancer treatment, multi-drug resistance is a serious clinical problem, underlining the need for further advances [29]. Therefore, new therapeutic solutions are being sought that are effective in the treatment of breast tumors that are not susceptible to conventional treatment regimens. Forthcoming challenges include finding optimal combinations and sequences of systemic therapies.

New active agents, which effectively and selectively eliminate BC cells and additionally overcome drug resistance and enhance anticancer properties of currently used chemotherapeutics without destroying healthy tissue, are of great importance [22,30]. Chemotherapy with cisplatin is mostly used as TNBC treatment [31]. However, drug resistance to standard treatments used in the therapy of the luminal subtype of BC has extended the use of CDDP in these types of BC. Unfortunately, CDDP or its derivatives are limited by the many side effects and the occurrence of CDDP resistance [30]. Therefore, combinations of conventional chemotherapeutic agents and new active compounds with documented, significantly lower toxicity are used to overcome these obstacles. In this context, numerous synthetic or natural chemical compounds, including HDIs, have been identified and have become an interesting class of active agents for cancer therapy [32]. 

In our previous studies, we demonstrated beneficial effects of combining VPA or SAHA with CDDP in native and Notch1-altered TNBC cells using advanced isobolographic method of assessing drug–drug pharmacological interactions [18,22]. The isobolography is a very rigorous pharmacodynamic method to establish the type of pharmacological interaction between two or more active agents that exhibit a broad range of doses. The isobolographic analysis allows us to precisely classify the observed interactions of drugs used in mixture at the fixed drug-dose ratio, usually 1:1. Theoretically, four types of interaction can be distinguished: synergism (supra-additivity), additivity, relative antagonism (sub-additivity), and absolute antagonism (infra-additivity) [18,22]. Unfortunately, this method is not very common in determination of the types of drug–drug interactions in cancer-related studies. Usually, only simple correlation methods between tested active agents are used, where only a limited number of chosen concentrations are selected [18].

It has been demonstrated that HDIs affect Notch signaling in tumor-stimulating or tumor-suppressive ways in the cell-dependent context. In hepatocellular carcinoma (HCC) cell lines, SAHA inhibited Notch signaling pathway; therefore, combination of SAHA and Notch inhibition can be a strategy for HCC treatment in the future [33]. Similarly to SAHA, VPA decreased Notch activity through downregulation of HES1 (HES family bHLH transcription factor) and upregulation of p21 and p63 tumor suppressors in HCC [34]. Moreover, VPA showed a similar effect in the multiple myeloma cells, where VPA inhibited cancer cell proliferation via downregulation of the Notch signaling pathway [35]. Interestingly, in other types of tumors, VPA was shown to stimulate Notch expression. For example, VPA upregulated Notch1 expression, inhibited cell cycle, and induced of apoptosis, both in vitro and in vivo, as well as suppressed expression of the neuroendocrine tumor markers [36,37]. Similarly, in small-cell lung cancer cells, VPA activated Notch signaling by an increase of Notch1, Notch target gene HES1, and p21 expression, which resulted in the inhibition of growth of cancer cells and cell cycle at G1 phase [38]. In a similar manner, VPA upregulated Notch1 and inhibited growth of cancer cells in vitro and in vivo in a mouse xenograft model in pulmonary carcinoid, gastrointestinal, and neuroblastoma models [39]. 

To date, there are no reports on the impact of SAHA or VPA on the level of Notch activity in luminal-type BC. In our previous study, we demonstrated that SAHA significantly decreased Notch1 gene expression in a dose-dependent manner in MDA-MB-231 TNBC cells. A similar tendency was observed for the combination of SAHA + CDDP. However, there was no statistically significant difference in Notch1 expression between control and VPA treatment individually or in combination with CDDP at the mRNA level in MDA-MB-231 cells [18]. In line with our previous work where high Notch increased the proliferation and survival of MDA-MB-231 cells [18], our study demonstrates that high Notch1 activity caused resistance to applied HDIs, while low or normal Notch activity sensitized cells for the HDIs applied separately and in combination with CDDP, showing that Notch1 activity influences treatment efficiency in luminal-type BC cells. Interestingly, combinational treatment of CDDP and HDIs sensitized Notch1^high^MCF7 to therapy. In our experiments, IC_50_ values for VPA and SAHA were two-fold lower in Notch1^low^MCF7 cells compared to those cells with Notch1^high^MCF7, whereas this kind of correlation was not seen in TNBC MDA-MB-231 cells [18]. The enhanced the cytotoxic effect and a better therapeutic response in MCF7 cells, comparing to TNBC-derived cells, may result from the fact that MDA-MB-231 cells, like other TNBC, are more resistant to therapy, resulting from heterogeneous cell populations and more malignant phenotype [40]. Moreover, MCF7 cells express HER2 receptor. It has been demonstrated that there is a direct connection between Notch1 and HER2 receptors. Co-immunoprecipitation and double immunofluorescence staining exhibited that Notch1 co-localizes and interacts with HER2 in HER2-positive BC cells. HER2 can reduce binding between Notch1 ligands and their receptors and, in consequence, decrease the activated level of Notch1 and influence breast cancer cells’ proliferation [41]. Both tested HDIs produce different epigenetic changes that affect the expression of several genes through inhibition of selected HDACs and increase in histone acetylation level [8]. Thereby, they can affect a set of genes differentially expressed in analyzed cell lines, resulting in varied response for the applied treatment. However, what is interesting in the case of MCF-7 cells observed in our studies, regardless of Notch1 expression, combined treatment of HDIs and CDDP resulted in favorable outcome by means of drug–drug interaction, showing additivity or additivity with tendency to synergy. Isobolographic analysis of the pharmacological interaction between CDDP and HDIs demonstrated that the mixture of CDDP with VPA at the fixed ratio of 1:1 exerted an additive interaction in both Notch1^high^MCF7 and Notch1^low^MCF7 cells. A similar tendency was observed in the native MCF7 cells co-treated with CDDP and VPA [22]. Interestingly, a better type of pharmacological interaction (additivity with tendency toward synergy) between CDDP and SAHA has been observed in the cells with altered Notch activity compared with the native cells (additivity) in MCF7 cells [22]. Antagonistic type of pharmacological interaction was not observed in the combination of HDIs and CDDP in any BC cell line. Therefore, these active compounds can be successfully used together in patients with altered Notch1 activity. Antagonistic interaction in terms of anticancer studies is not recommended because drugs should be used in higher doses to eliminate 50% of cancer cells. In such a case, under clinical conditions, patients should receive higher drug doses and, thus, adverse effects may occur more frequently than expected [42].

Obtained results are very promising, especially for patients with the more aggressive type of luminal BC with a high level of Notch1 activity. In our study, we observed that decreased Notch1 activity in luminal-type MCF7 cells results in the increased sensitivity of these cells for CDDP treatment, proving that measuring the prognostic value of Notch1 expression can help to guide individual therapy for BC patients [18]. 

## 4. Materials and Methods

### 4.1. Drugs

Suberoylanilide hydroxamic acid (vorinostat, SAHA) was purchased from Cayman Chemical (San Diego, CA, USA). Stock solution was prepared in dimethyl sulfoxide (DMSO) at 10-mM concentration. Valproic acid (VPA) and cisplatin (CDDP) were purchased from Sigma (St. Louis, MO, USA) and dissolved in phosphate buffered saline (PBS), (Sigma; St. Louis, MO, USA) with Ca^2+^ and Mg^2+^ at 100 mM and 1 mg/mL concentration as stock solutions, respectively. The drugs were diluted in order to obtain the final working concentrations using respective culture medium.

### 4.2. Cells

MCF7 (ATTC©HTB-22) BC cell line was obtained from the American Type Culture Collection (Manassas, VA, USA). BC cells were grown in DMEM/HAM F12 culture medium (Sigma, St. Louis, MO, USA) supplemented with 10% fetal bovine serum (FBS) (Sigma; St. Louis, MO, USA), 100 IU/mL of penicillin (Sigma; St. Louis, MO, USA), and 100 µg/mL of streptomycin (Sigma; St. Louis, MO, USA). Mycoplasma-free cultures were maintained at 37 °C in a humidified atmosphere with 5% CO_2_.

### 4.3. Transfection Procedure and Development of MCF7 Breast Cancer Cell Lines with Increased (Notch1^high^) and Decreased (Notch1^low^) Notch1 Activity 

Transfection procedure and development of MCF7 BC cell lines with increased (Notch1^high^) and decreased (Notch1^low^) Notch1 activity have been described previously [24]. 

### 4.4. Western Blotting Analysis and Luciferase Reporter Assay: Evaluation of the Effectiveness of the Transfection Procedure and Development of MCF7 Breast Cancer Cell Lines with Increased (Notch1^high^) and Decreased (Notch1^low^) Notch1 Activity 

Western blotting analysis and luciferase reporter assay as evaluation tests of the effectiveness of the transfection procedure and development of MCF7 BC cell lines with increased (Notch1^high^) and decreased (Notch1^low^) Notch1 activity have been described previously [24]. 

### 4.5. Cell Viability Assay 

MCF7 [22], Notch1^high^MCF7, and Notch1^low^MCF7 BC cells were platted on 96-well microplates at a density of 3 × 10^4^ cells/mL. The cells were incubated with VPA (10–1000 μg/mL), SAHA (0.02–3 μg/mL), or CDDP (0.01–10 μg/mL) for 96 h individually and in combination (VPA with CDDP; SAHA with CDDP). Then, the cells were incubated with the MTT [3-(4,5-dimethylthiazol-2-yl)-2,5-diphenyltetrazolium bromide] solution (5 mg/mL, Sigma; St. Louis, MO, USA) for 3 h. During this time, MTT was metabolized by living cells to purple formazan crystals, which were solubilized in a sodium dodecyl sulfate (SDS) buffer (10% SDS in 0.01 N HCl) overnight. The optical density of the product was measured at 570 nm using an Infinite M200 Pro microplate reader (Tecan, Männedorf, Switzerland). The results of combined treatment of CDDP and HDIs were analyzed according to the isobolographic protocol. The drug doses were determined based on the IC_50_ values.

### 4.6. Isobolographic Analysis of Pharmacological Interactions between HDIs and CDDP

Types of pharmacological interactions between HDIs and CDDP for MCF7, Notch1^high^MCF7, and Notch1^low^MCF7 BC cell lines were analyzed using the isobolographic analysis, as described previously [22,30]. The isobolography permits accurate classification of the pharmacological interactions of drugs used in the mixture at the fixed drug-dose ratio (mostly, 1:1). Four types of drug–drug interactions can be discerned: synergy (supra-additivity), additivity, relative antagonism (sub-additivity), and absolute antagonism (infra-additivity) [22]. To begin isobolographic analysis of interaction between CDDP and VPA or SAHA, the inhibition of cell viability of Notch1^high^MCF7 and Notch1^low^MCF7 cells was determined. From log-probit dose-response effects of VPA, SAHA, and CDDP in BC cell lines, median inhibitory concentrations (IC_50_ values) for the tested drugs were calculated, as advised earlier [22]. The type of drug–drug interactions between CDDP and VPA or SAHA was established by comparing the experimentally determined IC_50_ mix values (at the fixed-ratio of 1:1) with the theoretically calculated additive IC_50_ add values, according to the methods described elsewhere [22,25,30]. As the dose-response effects for VPA, SAHA, and CDDP in all investigated cell lines were non-parallel to one another, a type I isobolographic analysis for non-parallel dose-response effect curves was used, as advised earlier [22]. 

### 4.7. Statistical Analysis 

The data were analyzed using GraphPad Prism software (San Diego, CA, USA) with one-way ANOVA (Tukey post hoc testing). Results were presented as mean ± standard error of the mean (± S.E.M.) and p < 0.05 was considered to indicate a statistically significant difference. Log-probit analysis was used to determine the experimentally derived IC_50_ and IC_50_ mix values for VPA, SAHA, and CDDP when the drugs were administered individually or in combination for the fixed ratio of 1:1. Statistical difference between the experimentally derived IC_50_ mix values and the theoretically calculated additive IC_50_ add values (for lower and upper line of additivity) was assessed with unpaired Student’s t-test, as presented elsewhere [22]. 

## 5. Conclusions

In summary, combinational treatment of CDDP with VPA or SAHA sensitizes MCF7 luminal BC cells with high Notch1 activity to therapy. Moreover, our isobolographic analysis of pharmacological drug–drug interactions demonstrated that treatment of CDDP with HDIs could be used to optimize a combined therapy based on CDDP against Notch1-altered luminal MCF7 cells. Considering the fact that CDDP induces multiple adverse effects, the use of less toxic doses of this standard chemotherapeutic in combination with clinically available doses of HDIs seems to be an interesting therapeutic approach. If pre-clinical model, including xenografts, as well as clinical randomized trials prove the in vivo efficacy of these combinations, they might be able to reduce dose-dependent side effects, increase the overall survival of BC patients, and improve quality of their lives.

## Figures and Tables

**Table 1:** IC_50_ values (µg/mL) for cisplatin (CDDP) and two histone deacetylase inhibitors (HDIs): vorinostat (SAHA) and valproic acid (VPA) in native [22] and MCF7 cells with Notch1 variations.

Cell Line	CDDP	SAHA	VPA
Notch1^high^MCF7	4.554	1.052	479.4
MCF7 (native)	2.495	0.746	465.68
Notch1^low^MCF7	3.557	0.474	204.2

**Table 2:** Type I isobolographic analysis of interactions (for non-parallel DRRCs) between CDDP and SAHA or VPA at the fixed-ratio combination of 1:1 on MCF7 with increased or decreased activity of Notch1. Results are presented as median inhibitory concentrations (IC_50_ values in μg/mL ± S.E.M.) for two-drug mixtures, determined either experimentally (IC_50_ mix) or theoretically calculated (IC_50_ add) from the equations of additivity [26,27], blocking proliferation in 50% of tested cells in cancer cell lines (MCF7 with increased or decreased activity of Notch1). The n_mix_—total number of items used at those concentrations whose expected anti-proliferative effects ranged between 16% and 84% (i.e., 4 and 6 probits) for the experimental mixture; n_add_—total number of items calculated for the additive mixture of the drugs examined; L-_IC50 add_ value calculated from the equation for the lower line of additivity; U-_IC50 add_ value calculated from the equation for the upper line of additivity. Statistical evaluation of data was performed with unpaired Student’s t-test.

Cell Line	Combination	IC50 mix (μg/mL)	n_mix_	^L^ IC_50 add_ (μg/mL)	n_add_	^U^ IC_50 add_ (μg/mL)	n_add_
Notch1^high^MCF7	CDDP+SAHA	0.572 ± 0.362	24	0.759 ± 1.084	56	4.846 ± 1.970	56
Notch1^high^MCF7	CDDP+VPA	48.87 ± 27.65	30	169.9 ± 85.13	56	314.4 ± 114.0	56
Notch1^low^MCF7	CDDP+SAHA	1.262 ± 0.737	24	1.497 ± 1.180	56	2.545 ± 1.522	56
Notch1^low^MCF7	CDDP+VPA	38.04 ± 15.14	30	83.75 ± 45.30	56	123.8 ± 55.30	56

**Table 3:** Isobolographic analysis of interactions between CDDP and SAHA or VPA at the fixed-ratio combination of 1:1 on cells with increased or decreased activity of Notch1 with reference to native MCF7 cells [22].

Combination	Notch1^high^MCF7	MCF7	Notch1^low^MCF7
CDDP+SAHA	additivity	additivity	additivity
CDDP+VPA	additivity with tendencytowards synergy	additivity	additivity with tendencytowards synergy

## Data Availability

Data available in a publicly accessible repository.
